# South West Paediatric Club, Meeting in Bristol, October, 1987

**Published:** 1989-02

**Authors:** 


### South West Paediatric Club

### Meeting in Bristol, October 1987

### LOOKING BACK ON 40 YEARS OF PAEDIATRICS

**Bernard M Laurance MB FRCP DCH**
Consultant Paediatrician (retired)
Lately at the Queen Elizabeth Hospital for Children, London

An anecdotal account of paediatrics from 1947 when there were approximately 100 members of the British Paediatric Association (BPA) compared with over 2000 now. When a Houseman, was first described; fibrocystic disease and starch was excluded from all food for coeliac children resulting in dramatic improvement. Pink disease was also common and only years later was mercury in teething powders recognised as the cause. Each morning began with 6 intrathecal injections of the newly discovered streptomycin. 1947 was one of the worst epidemic years for poliomyelitis. Visiting of children in hospital was barbarically restricted to 2 hours on Sundays only.

#### The Newborn

Routine examinations of the newborn were denied to paediatricians who were only allowed to see those babies the midwives considered necessary. Haemorrhagic disease of the newborn was treated with injections of 10 ml of fresh blood which was usually the fathers. Replacement transfusions for rhesus incompatibility were marathons in time as well as effort in pushing blood through narrow bore, long, hard polyvinyl tubes.

Junior staff were expected the wait for the arrival of their chiefs in the front hall of hospitals—often long after the appointed time. On one occasion mine arrived with a bottle of Royal wee for testing because of PUO; subsequently proved to be measles.

Professor Neale was my mentor in Bristol. He brimmed over with energy and new ideas. A great supporter of junior staff.

Retrolental fibroplasia was recently described but the discovery that oxygen was the culprit had yet to happen.

My first Consultant post was in Derby where I joined the renounned Dr (later Sir) Douglas Hubble who later moved to the Chair of Paediatrics in Birmingham.

A new post entailed my innovations services for the handicapped where in their infancy. There was also the struggle to achieve "rooming in" of babies with their mothers in the lying-in wards. For the premature babies I continued the Bristol practice of giving Vitamin K 10 mg tds. To my consternation, 6 babies died in as many weeks of kernicterus. Only after this was the association of the two recognised. Much time was preoccupied with replacement transfusions, meningomyeloceles and other chronic problems. Steroids had just become available and it was some time before their proper use was understood. Prior to this, treatment of nephrotics was by drainage of oedema fluid from the dorsa of the feet after pricking them with needles. Even applying malarial mosquitoes to produce fever was successful sometimes.

Secondment to the Chair of Paediatrics at Makerere University, Uganda for two and a half years gave an insight into the problems of immigrants and their diseases. It also gave wider experience of administration of paediatric services. This interlude proved valuable for teaching under- and postgraduates at the Queen Elizabeth Hospital for Children in London. In a different but allied sphere, I served as Honorary Secretary to the BPA.

Paediatrics has made tremendous strides in the last 40 years. I would still opt to be a paediatrician given the chance to start again.

### ULTRASOUND EXAMINATION OF THE INFANT HIP

**N. M. P. Clarke, Ch.M., F.R.C.S.**
Department of Orthopaedic Surgery, Bristol Royal Infirmary, Bristol

Clinical examination as a method of screening for congenital dislocation of the hip (CDH) at birth remains the most efficient screening method. Recently, however, ultrasound examination of the infant hip has shown to be a reliable method for delineating dysplasia and displacement of the femoral head. This method is now used extensively in the diagnosis and management of neonatal hip instability. Ultrasound has several advantages. It is non-invasive and may be repeated as many times as is necessary. Additionally, a dynamic examination can be performed using real time ultrasound and thus the specificity may be conferred upon a tactile, subjective interpretation of, for instance, a clicking hip.

Studies have shown that ultrasound may allow the clinician to detect those unstable hips which will resolve spontaneously and thus avoid unnecessary treatment of hip instability. Clearly, this is a great advantage since splinting of neonatal hips does have a certain morbidity. Current controversies, however, are directed towards the use of ultrasound as a screening method for the detection of hip displacement in the neonate. There are problems with the reproducibility of the technique and observer error and certainly early in the learning curve the examiner may have a high false positive rate for abnormality in the neonate. A study has shown that the screening of "at risk" infants, however, does not reproduce the incidence of the late cases of CDH and therefore it would appear that ultrasound should be used in all infants rather than in a select group. This, of course, will depend on the adequacy of resources and the availability of equipment and examiners. At present ultrasound examination of the infant hip is certainly indicated in "at risk" infants but also in the early management of congenital displacement of the hip. However, before screening with ultrasound can be commenced, the ultrasound technique will need to be standardised and resources allocated.

### MENINGOCOCCAL DISEASE IN GLOUCESTERSHIRE

**Dr James M. Stuart**
Senior Registrar in Community Medicine, Gloucestershire

#### The Outbreak

The winter of 1981/82 marked the beginning of an outbreak of meningococcal disease in Gloucester Health District (population c. 300,000). The outbreak was identified by a rising attack rate of disease, mainly due to a sulphonamide resistant Group B type 15 meningococcus (B15R) which was new to the district. Between 1st October 1981 and 31st August 1987 there were 98 cases of meningococcal meningitis and/or septicaemia, an average incidence of 5.4/100,000 per annum. This compares with an average national notification rate of about 1/100,000 per annum.

**Particular Features**

1. After 6 years the outbreak is still continuing. There were 30 cases in 1986 due in part to a concomitant rise in Group C disease.
2. 65% of the cases have been confined to the local authority district of Stroud (population c. 100,000).
3. The attack rate has been highest in teenagers, 55% of those affected being between the ages of 10 and 19.
4. Community carrier rates of the B15R strain, where investigated, have been low, (less than 1%).
5. The case fatality rate (7%) has been slightly lower than expected.
6. Official notifications have remained about half the number of locally ascertained meningitis cases throughout the outbreak. (Septicaemia alone is not statutorily notifiable).
7. Media interest has been considerable.

### HYPOXIA AND THE BRAIN

**Professor I. A. Silver**
Department of Pathology, Medical School, Bristol

During hypoxia/ischaemia energy loss in brain is rapid due to lack of ATP and glycogen reserves and reliance on glucose and $O_2$ delivery. Neurones survive longer in hypoxia despite inadequate $O_2$ because high bloodflow brings glucose for glycolysis and removes toxic metabolites e.g. lactate. 'High flow' ischaemia provides some glucose with inadequate washout; lactate accumulates and pH falls. 'Low flow' ischaemia gives less cellular acidosis due to lack of substrate. Reoxygenation or restoration of bloodflow may produce $O_2$ radicals which can cause membrane damage especially when high $O_2$ concentrations are used in resuscitation. Energy loss causes failure of the (plasma and mitochondrial) membrane ion pumps ($Na^+$, $K^+$ and $Ca^{++}$ ATPase). There is loss of intracellular $K^+$ which is exchanged for $Na^+$ together with water and later with $Ca^{++}$. Fetal brain is relatively resistant to ion leakage and low body temperature is protective. CNS co-ordination is lost with depressed synaptic transmission; then membrane potential; then massive ion leakage; then irreversible changes. Permanent damage may be due to raised intracellular calcium. Initially, $[Ca^{++}]_i$ rises due to release from internal stores; later there is leakage in from CSF through a variety of 'channels'. One, found in hypoxia sensitive areas, is opened by transmitters such as aspartate. This can be blocked by ketamine which may be useful therapeutically. Other $Ca^{++}$ channel blockers have not been found so effective.

### PASTIMES IN PERINATAL PATHOLOGY

**Peter M Dunn**
Professor of Perinatal Medicine and Child Health, University of Bristol

As a neonatal registrar between 1959 and 1968 I was able, with the kind permission of Dr Hans Kohler and Dr Norman Brown, to undertake postmortem examination of some hundreds of newborn babies that had died soon after birth. My purpose is to discuss some of my findings and to try to communicate the interest and value these studies had in providing an insight into the pathology and aetiology of a number of neonatal conditions and also in aiding clinical management. In an abstract such as this it is only possible to list the main subjects to be discussed;

(a) the methodology and clinical importance of localising the tip of catheters passed up the umbilical vessels;
(b) the occurrence and significance of liver damage in infants born with severe haemolytic disease;
(c) the embryology, anatomy and pathology of congenital dislocation of the hip;
(d) the role of oligohydramnios due to either fetal oliguria or premature rupture of the membranes in causing compression deformities and also pulmonary hyperplasia;
(e) the pathology of congenital sternomastoid torticollis and congenital postural scoliosis;
(f) the frequency and importance of pneumothorax and pneumomediastinum in the neonatal period.

This experience led me to appreciate the wisdom of Dr James Blundell's advice to medical students of Guy's Hospital in the 1820's:

"Beware of temerity—*see what can be done on the dead body*—gather facts—form inferences—write little—meditate much".

### CIRCULATORY ADAPTATION IN THE NEONATAL BRAIN

**M. R. Drayton**
Senior Registrar in Neonatal Paediatrics, Bristol

Ultrasound scanning has revealed that the most important determinants of neurological damage in preterm infants are intracerebral haemorrhage and more importantly cerebral ischaemia, and that these twin pathologies are extremely common in the first few days of life but relatively rare thereafter. This time-course suggests that the enormous circulatory adaptation from intra to extrauterine life may be aetiologically important. Previous Doppler ultrasound studies of the intracerebral arteries are likely to have been confounded by changing calibre of these vessels themselves. We have shown that even in preterm infants the vessels have a well developed muscular wall containing contractile protein and are innervated. A new Doppler method of assessing volumetric flow to the head, about 80% of which is believed to supply the bran has been developed. Studies during the first 48 hours of life in preterm infants show that while blood velocity in the intracerebral arteries approximately doubles, cerebral perfusion apparently remains constant. It is concluded that the large intracerebral arteries are vasodilated at birth becoming progressively constricted perhaps as arterial blood pressure rises. The pathophysiological, and clinical implications of these observations are discussed.

### RENAL FUNCTION AND ITS MEASUREMENT IN THE NEWBORN

**Dr B H Wilkins**
Research Fellow and Senior Registrar, Southmead Hospital, Bristol

Renal function is very difficult to measure in the newborn in routine practice. Creatinine cannot be mesured reliably because of interfering substances in plasma such as bilirubin. In any case plasma creatinine rises only slowly after the onset of acute renal failure.

It has often been stated that renal function is diminished in the newborn and that this causes a reduced ability to excrete water, salt and waste products of metabolism. This view has been based on measurements of renal function using methods applicable to adults.

We have measured renal function in 40 very low birthweight newborn infants using insulin clearance methods which have been refined for use in this group of subjects. Glomerular filtration rate varies from 0.5 to 1.0 ml/min/kg and is no different in even the sickest and smallest babies. Considering the low metabolic rate of these babies, these values are quite adequate for excretory purposes.

Tubular function, however, is compromised in sick immature infants causing excessive sodium wasting, some babies excreting as much as a fifth of their total body sodium per day. We may speculate, therefore that GFR may actually be too high in that the immature tubules are flooded with more filtrate than can be reabsorbed.

*Continued on page 32a.*

### 3. South West Paediatric Club

*Continued from page 30.*

### PAEDIATRIC DERMATOLOGY

**Dr Cameron Kennedy**
Consultant Dermatologist, Bristol

In Great Britain skin disorders in children are managed in the hospital by either paediatricians or dermatologists. There are only two full time paediatric dermatologists in this country and they are not fully trained in paediatrics. This is in contrast to North America and Europe, where there are many doubly trained specialists. An important new develolpment has been the formation of the British Society for Paediatric Dermatology, the group that has a number of paediatricians as members, and next year there will be the first combined meeting with the British Paediatric Association.

A dermatologist working in a paediatric unit makes contributions to the diagnosis of a wide variety of disorders—some with important genetic and metabolic implications, and will be able to bring the breadth of dermatological expertise to managing common conditions such as atopic dermatitis and psoriasis.

The disorders discussed include the prenatal diagnosis of epidermolysis bullosa, neonatal lupus erythematosus, allergy and atopic dermatitis, fifth disease and human parvovirus, skin disease and sexual abuse, and skin changes in the immunosuppressed.

